# Current Trends in Food Health and Safety in Cross-Cultural Sensory and Consumer Science

**DOI:** 10.3390/foods10050965

**Published:** 2021-04-28

**Authors:** Derek Victor Byrne

**Affiliations:** 1 Food Quality Perception and Society Science Team, iSENSE Lab, Department of Food Science, Faculty of Technical Sciences, Aarhus University, Agro Food Park 48, DK-8200 Aarhus N, Denmark; derekv.byrne@food.au.dk; 2 Food & Health Research, Sino-Danish Center (SDC), Niels Jensens Vej 2, DK-8000 Aarhus C, Denmark

## 1. Introduction

The global food and food technology market is in rapid growth, and food investment is central in many governments’ growth plans. A core focus can be said to be on the food industry’s ability to maintain and strengthen its position and exploit the unique opportunities for export-driven growth, especially in the export of high-quality food with relevant sensory health, safety, and sustainability properties as key elements. Thus, the concept of cross-cultural perspectives in research in food per se is critically important, none more so than in relation to the human perception area in food and health. Food concepts are very different, of course, in different jurisdictions, with respect to different markets and cultures having very different perspectives on what is considered a palatable, acceptable, or useful food or food product. In simple terms, one size does not fit all in the majority of cases.

Cross-cultural studies have been in focus for some time in the food space and in particular in relation to food design via the senses and from a consumer-driven perspective. From one of the earliest overviews by Prescott and Bell (1995) [1], which reviewed the literature on basic cross-cultural determinants of food acceptability focusing on chemosensory perceptions and preferences from the point of view of their ability to explain differences in food selection in different cultures [1]. On to one of the latest perspectives by Rodrigues et al. (2019) on consumers’ food decisions and eating habits as well as cross-cultural eating focusing on the application of virtual reality, mobile applications, and social media, amongst other areas [2]. It is, of course, the case that the space over the last 25 years up to the present anthology collection in “Food, Health and Safety in Cross-Cultural Consumer Contexts” is peppered with a collection of works addressing perspectives linked to the senses, and cross-cultural applicability of note would be, e.g., linking the senses to psychology [3], cross-cultural differences in cross-modal correspondences [4], and measurement of food preference and reward in cross-cultural contexts [5], to consumers’ associations with wellbeing in a cross-cultural studies [6].

Specific areas of research presented as relevant for the scope of this Special Issue indicate, clearly, the ever-widening area of cross-cultural research with respect to sensory and consumer science regarding food and health. Areas that were in focus for the special issue and cross-cultural research were as follows. (1) Food quality, processing, and production: focusing on understanding food processing, quality, and perception via a synergy of multisensory human food analysis, combined with novel and sustainable production techniques. (2) Microbial food safety and hygiene: dealing with microbial food safety behaviors and focusing on the knowledge to detect foodborne pathogens, sources of outbreaks of foodborne diseases, and novel strategies to ensure food safety for the consumer. (3) Food business, marketing, and the consumer: focusing on research on the development, marketing, and distribution of foods to generate insight into consumer behavior for the benefit of food industries and public policy. (4) Food economics and the supply chain: dealing with research in logistics and supply chain management regarding the concepts around economic thinking in food production, trade, and the management of food quality and safety across the supply chain. (5) Food sociology and eating: the sociological elements of food safety and quality, including research around the social and cultural aspects of eating, production, and new technologies, as well as the role of legal frameworks and regulations. (6) Nutrition and health: focusing on the effects on health of specific food, food components, and supplements in health and disease prevention, the rationale for nutritional recommendations, and food and nutrition security.

The present Special Issue’s focus overall was food, health, and safety in cross-cultural consumer contexts” for innovative food solutions to meet global food challenges, which can be best addressed by research-based synergies linking, e.g., different jurisdictions, countries, and continents in the food area.

Ultimately, in this special issue we have included contributions that encompass key current research on food, health and safety in cross-cultural consumer contexts with respect to food science synergies for sustainable, healthy, and high-quality food supply, security, and consumption scenarios across the entire food chain from “farm to fork” in cross-cultural contexts. Specifically, we have brought together articles that encompass the wide scope of cross-cultural multidisciplinary research as alluded to above with perspectives in the space related to the determination of the key factors involved. The articles included can be considered to cover stakeholders in cross-cultural perception, from the senses, with respect to differences in sensitivity [7,8,9], on to consumer preferences [10,11,12], food pleasure and appetite [13,14], perception of food quality and safety [15,16,17], and finally key factors in relation to consumer adoption and label information in the market itself [18,19]. This collection of articles is in essence a snapshot of the wide focus and general relevance of sensory and consumer science in cross-cultural studies in food health and safety and we hope it inspires researchers to consider this very interesting and ever-growing space in their future work.

## 2. A Synopsis of Special Issue Research

### 2.1. Sensory Differences

Thus, with respect to sensory differences, Junge et al. (2020) performed research in the area of sweetness and sour interaction [7]. The authors indicated that tastes interact in almost every consumed food or beverage, yet many aspects of interactions, such as sweet–sour interactions, were not well understood. The study investigated the interaction between sweetness from sucrose and sourness from citric and tartaric acids, respectively, in a cross-cultural consumer study conducted in China and Denmark. Overall, it was determined that culture did not impact the suppression of sweetness intensity ratings of citric or tartaric acids, whereas it did influence sourness intensity ratings. While the Danish consumers showed similar suppression of sourness by both acids, the Chinese consumers were more susceptible toward the sourness suppression caused by sucrose in the tartaric acid–sucrose mixture compared to the citric acid–sucrose mixture. These results indicated that individual differences in taste perception might affect perception of sweet–sour taste interactions, at least in aqueous solutions.

Moreover, in relation to sensory differences, authors Nóbrega et al. (2020) looked at two segments within the rather large Brazilian food service industry with respect to bestselling coffees and serving temperatures with respect to health and safety [8]. The serving temperatures of best-selling coffee beverages in 50 low-cost food service establishments (LCFS) and 50 coffee shops (CS) were studied. The bestsellers in the LCFS were dominated by 50 mL shots of sweetened black coffee served in disposable polystyrene (PS) cups from thermos flasks. In the CS, 50 mL shots of freshly brewed espresso served in porcelain cups were the dominant beverage. The serving temperatures of all beverages were on average 90% and 68% above 65 °C in the LCFS and CS, respectively. Furthermore, the cooling periods of hot water systems were investigated. When median temperatures of the best-selling coffees are considered, consumers should allow a minimum cooling time before drinking of about 2 min at both LCFS and CS. Nóbrega et al. (2020) concluded that further studies to complete a nationwide picture of coffee consumption habits and the temperature at which consumption commonly occurs in Brazil could also present an excellent opportunity for an esophageal cancer risk assessment for hot coffee beverage consumption.

Lastly, in a study of texture preference of Chinese, Korean, and US consumers by Wong et al. (2020) [9], the authors aimed to understand the drivers of liking dried apple and pear chips with various textures. The possibility of hedonic transfer from snack texture preferences to fruit-chip texture preferences was also investigated among Chinese and Koreans. Consumers rated their level of liking for each sample and then they performed hedonic-based projective mapping with the same samples. In the hedonic texture transfer investigation, consumers rated their acceptance of nine snacks with various textures but possessing similar textures to those of dried fruit samples. Most consumers disliked samples with a soft or jelly-like texture and liked samples with a crispy texture. Cross-cultural differences were observed in the liking of puffy samples, with both Chinese and Koreans liking puffy samples as much as crispy ones for their melting characteristics in the mouth, while US consumers perceived the puffy samples as being Styrofoam-like and disliked them. Hedonic transfer was observed from snack texture preferences in fruit chips. Individual texture preferences for snacks seemed to significantly affect the texture preferences for fruit chips. Wong et al. (2020) concluded that the overall impact of the study was the potential to predict the potential market in the chosen countries using hedonic-based projective mapping.

### 2.2. Consumer Preferences

In relation to choice, per se, in cross-cultural contexts, Profeta et al. (2021) looked at consumer preferences for meat hybrids (referred to as meathybrids) in Germany and Belgium [10]. The authors’ basis for the study was high levels of meat consumption are increasingly being criticized for ethical, environmental, and social reasons. Plant-based meat substitutes have been identified as healthy sources of protein that, in comparison to meat, offer a number of social, environmental, and health benefits and may play a role in reducing meat consumption. In meathybrids, only a fraction of the meat product (e.g., 20% to 50%) is replaced with plant-based proteins. Profeta et al. (2021) demonstrated that in many countries, consumers are highly attached to meat and consider it as an essential and integral element of their daily diet. For consumers that are not interested in vegan or vegetarian alternatives as meat substitutes, meathybrids could be a low-threshold option for a more sustainable food consumption behavior [10]. The authors showed that more than fifty percent of consumers substitute meat at least occasionally. Thus, about half of the respondents reveal an eligible consumption behavior with respect to sustainability and healthiness, at least sometimes. The applied discrete choice experiment demonstrated that the analyzed meat products are the most preferred by consumers. Nonetheless, the tested meathybrid variants with different shares of plant-based proteins took the second position followed by the vegetarian-based alternatives. Therefore, meathybrids could facilitate the diet transition of meat-eaters in the direction toward a more healthy and sustainable consumption. The analyzed consumer segment was more open-minded to the meathybrid concept in comparison to the vegetarian substitutes [10].

Further to consumer preference in the cross-cultural space, Garvey et al. (2020) looked at perception and liking among Irish, German, and US consumers for salted butter produced from different feed systems [11]. Overall, it was presented there was no significant difference in overall liking of the butters among any of the consumers, although cross-cultural preferences were evident. Sensory attribute differences based on animal diet were evident across the three countries, as identified by German and Irish assessors and trained US panelists, which were likely influenced by familiarity. Of volatiles measured, Garvey et al. 2020 indicated that the abundance of specific volatile aromatic compounds, especially some aldehydes and ketones, were significantly impacted by the feed system and may also contribute to some of the perceived sensory attribute differences in these butters.

Additionally, in relation to preference in relation to a protected geographical indication (PGI) product in the European Union, authors Kelly et al. (2020) investigated the PGI product called Waterford Blaa, which is a bread product specific to Ireland’s East Cost, traditionally [12]. This study aimed to determine whether cultural background/product familiarity, gender, and/or age impacted consumer liking of three Waterford Blaa products and explored product acceptability between product-familiar and product-unfamiliar consumer cohorts in Ireland and the UK, respectively. Familiarity with Blaa impacted consumer liking, particularly with respect to characteristic flour dusting, which is a unique property of Waterford Blaa. UK consumers felt that all Blaas had too much flour dusting. Flavor was also important for UK consumers. Irish consumer liking was more influenced by the hardness of the Blaas, with harder products being less preferred. Age and gender did not impact liking for Blaas within Irish consumers, but gender differences were observed among UK consumers, males liking the appearance significantly more than females. In cross-cultural contexts, such PGI products which have largely fixed formats and properties, it is critical to determine if they can at all cross borders in terms of sensory and consumer acceptance or will they simply be niche, linked to only diaspora or food-curious individuals.

### 2.3. Appetite Pleasure and Ingestion Sensations

Another area that has emerged of late as a focus for cross-cultural food design is the area of a food’s influence on appetite through pleasure and ingestion sensations. In the present volume, Duerlund et al. (2020) investigated post-ingestive sensations and how they drive perceived food pleasure [13]. The authors aimed to compare Chinese and Danish consumers in their post-ingestive drivers of post-ingestive food pleasure (PIFP). Duerlund et al. (2020) define PIFP as a “subjective conscious sensation of pleasure and joy experienced after eating”. Key results revealed perceived satisfaction as well as mental, overall and physical wellbeing to be highly influential on PIFP in both countries. Moreover, Danish consumers perceived appetite-related sensations, such as satiety, hunger, desire-to-eat and in-need-of-food, to be influential on PIFP, which was not the case in China. In China, more vitality-related sensations, such as energized, relaxation and concentration, were found to be drivers of PIFP. These results suggest similarities but also distinct subtleties in the cultural constructs of PIFP in Denmark and in China. Duerlund et al. (2020) overall suggested that focusing on food pleasure as a post-ingestive measure provides valuable output, deeper insights into what drives food pleasure, and, importantly, takes us beyond the processes only active during the actual eating event.

Furthermore, in the present volume, Laaksonen et al. (2020) in Finnish and Chinese contexts looked at oat product concepts [14]. The authors present that oats and oat-based products were increasingly popular among consumers and the food industry, and whilst studies exist on the sensory characteristics of oats as such, previous studies focusing specifically on the pleasantness of oats, and especially investigations of a wide range of oat products eaten by European and Asian consumers, are scarce. A questionnaire revealed that Finnish consumers rated the pleasantness and familiarity of several oat product categories, such as breads and porridges, higher compared to participants from other cultures. Further, Laaksonen et al. 2020 indicated that sensory tests showed both similarities, e.g., porridges were described as “natural,” “healthy,” and “oat-like,” and differences between countries, e.g., sweet biscuits were described as “crispy” and “hard” by Finnish consumers and “strange” and “musty” by Chinese consumers. Sweet products were unanimously preferred. Moreover, authors indicated that the culture had an important role affecting the rating of pleasantness and familiarity of oat product categories, whereas food neophobia and health interest status also had an influence [14].

### 2.4. Perception of Quality and Safety

In relation to food safety and quality, authors Haas et al. (2021) investigated perception differences in the western Balkans [15]. The authors present that domestic food markets are of significant importance to Kosovar and Albanian companies because access to export markets is underdeveloped, partly as a result of the gaps in food safety and quality standards. Identifying Kosovar and Albanian consumers’ use of food safety attributes and an evaluation of the quality of domestic food versus imported food were the research objectives of this study. Haas et al. 2021 concluded that despite the prevalent problems with food safety, consumers in both countries considered domestic food to be safer as well as of higher quality than imported products. Kosovars were more likely than Albanians to perceive domestic food products to be significantly better than imported products. Female and better-educated consumers used information related to food safety more often. Expiry date, domestic and local origin, and brand reputation were the most frequently used safety and quality cues for both samples. International food standards, such as ISO or HACCP, are less frequently used as quality cues by these consumer groups. Haas et al. (2021) concluded that though this is a good result for these nations internally to have these perceptions, it is important to strengthen the institutional framework related to food safety and quality following best practices from EU countries, which could ultimately perhaps enable further development of export markets [15].

Moreover, in this special issue volume, Wang and Yueh (2020) investigated the area of food safety cognition with respect to consumer behavior, comparing students in Taiwan and mainland China [16]. The purpose of the study was to investigate how optimistic bias, consumption cognition, news attention, information credibility, and social trust affect the purchase intention of food consumption. Results showed that Taiwanese college students did not display optimistic bias, but Chinese students did. The models showed that both Taiwanese and Chinese students’ consumption cognition significantly influenced their purchase intentions, and news attention significantly influenced only Chinese students’ purchase intentions. The results revealed that optimistic bias can be reduced in different social contexts. This study also confirmed that people had optimistic bias on food safety issues based on which recommendations were made to increase public awareness of food safety as well as to improve the government’s certification system [16].

In relation to the area of high-risk food handling behaviors and consumer perception of food safety issues in these contexts, Cho et al. (2020) investigated behaviors and risk perceptions across time in South Korea among primary food handlers [17]. The authors gathered data in 2010 and 2019, and present that 2010 was characterized by a consumers’ risk perception–behavior disconnect, that is consumers believed they knew very well what the safest methods for food handling were, but responses regarding their behaviors did not support their confidence in the actual safety of their food. Such that consumers did not wash/trim foods before storage, thawed frozen foods at room temperature, and exposed leftovers to danger zone temperatures. Interestingly, these three particular trends were found to be similar when assessed again in 2019. The year 2010 was also characterized by other common high-risk behaviors: 70.0% of consumers divided a large portion of food into smaller pieces for storage, but few consumers (12.5%) labeled divided foods with relevant information, and they excessively reused kitchen utensils. Whereas in 2019, more consumers (25.7%) labeled food and usage periods for kitchen utensils were shortened. Consumers usually conformed to general food safety rules in both 2010 and 2019 in the following ways: separate storage of foods, storage of foods in the proper places for the proper periods, washing fruits/vegetables before eating, washing hands after handling potentially hazardous foods, and cooking foods and reheating leftovers to eat. Cho et al. 2021 concluded that their findings provided resources for understanding consumers’ high-risk behaviors/perceptions at home, highlighting the importance of behavioral control.

### 2.5. Consumer Adoption and Label Information

In this final section, we look at research included on consumer adoption and labeling. Nathan et al. (2021) indicated as a backdrop in the organic labeling area that in order to meet the rising global demand for food and to ensure food security in line with the United Nations Sustainable Development Goal 2, the elimination of hunger, technological advances have been introduced in the food production industry [18]. The organic food industry has benefitted from advances in food technology and innovation. However, there remains skepticism regarding organic foods on the part of consumers, specifically on consumers’ acceptance of food innovation technologies used in the production of organic foods, and this can be extrapolated to different cultural contexts. In the present volume, Nathan et al. (2021) measured factors that influence consumers’ food innovation adoption and subsequently their intention to purchase organic foods. Organic foods purchase behavior of Malaysian and Hungarian consumers was compared to examine differences between Asian and European consumers. The findings showed food innovation adoption as the most crucial predictor for the intention to purchase organic foods in Hungary, while the social lifestyle factor was the most influential in Malaysia. Other factors, such as environmental concerns and health consciousness, were also examined in relation to food innovation adoption and organic food consumerism. Overall, Nathan et al. (2021) present key differences between European and Asian organic foods consumers and provided recommendations for stakeholders interested in these markets going forward [18].

The remaining article in our special issue by Magalhaes et al. (2021) profiles knowledge, utility, and preference for beef traceability labeling between Spain and Brazil [19]. The consumer environment determined consumers’ buying behavior and product preferences, and understanding these factors would allow businesses in the industry to identify market demands. The authors contended that in both countries there were existing differences in the consumption of beef, in the production and the regulatory process concerning beef, and, in particular, in relation to the traceability systems. Having a traceability system is in fact mandatory in Spain and voluntary in Brazil. From these perspectives, Magalhaes et al. (2021) carried out a cross-cultural study through a self-administered questionnaire aimed at comparing and understanding familiarity with bovine traceability systems and traceability information on labels as a food security indicator. The authors concluded that traceability information was well received by consumers as an attribute of credibility, and consumers were interested in ensuring that the item they buy is of known and reliable origin. However, the authors contended that more incentives may help clarify the advantages of purchasing food with certified traceability, making it more effective for consumers to use this knowledge in different jurisdictions [19].

## 3. Conclusions

Overall, the research included in this volume covers a wide range of studies, from fundamental research to market applicability, with respect to sensory and consumer studies in food health and safety contexts. Groupings of the studies have been made to point out the diverse and core need to consider the human senses, consumer preferences and perception, across the food stakeholder chain in cross-cultural research. Several of the studies noted the need for more knowledge in their specific spaces and contended that this will lead to better-positioned food and food products when looking at world markets. An overall conclusion with respect to this collection would be that the human senses, consumer acceptance, and preferences are core to future food design with respect to understanding human perception of key aspects critical to the success of food transfer in modern cross-cultural contexts.
